# Chrysin, Apigenin and Acacetin Inhibit Tumor Necrosis Factor-Related Apoptosis—Inducing Ligand Receptor-1 (TRAIL-R1) on Activated RAW264.7 Macrophages

**DOI:** 10.3390/ijms150711510

**Published:** 2014-06-27

**Authors:** Monika Warat, Ewelina Szliszka, Ilona Korzonek-Szlacheta, Wojciech Król, Zenon P. Czuba

**Affiliations:** 1 Chair and Department of Microbiology and Immunology, Medical University of Silesia in Katowice, Jordana 19, 41-808 Zabrze, Poland; E-Mails: monika@warat.pl (M.W.); eszliszka@sum.edu.pl (E.S.); wkrol@sum.edu.pl (W.K.); 2 Department of Toxicology and Health Protection, Toxicology and Drug Addiction Division, Medical University of Silesia in Katowice, Medyków 18, 40-752 Katowice, Poland; E-Mail: ikorzonek@sum.edu.pl

**Keywords:** chrysin, apigenin, acacetin, TRAIL (tumor necrosis factor-related apoptosis-inducing ligand)-receptor (TRAIL-R) expression, RAW264.7

## Abstract

Expression level of Tumor Necrosis Factor—related apoptosis—inducing ligand (TRAIL) receptors is one of the most important factors of TRAIL-mediated apoptosis in cancer cells. We here report for the first time data concerning TRAIL-R1 and TRAIL-R2 receptor expression on RAW264.7 macrophages. Three substances belonging to flavones: chrysin, apigenin and acacetin which differ from their substituents at the 4' position in the phenyl ring were used in assays because of the variety of biological activities (e.g., anticancer activity) of the polyphenol compounds. The expression of TRAIL-R1 and TRAIL-R2 death receptors on non-stimulated and LPS (lipopolysaccharide)-stimulated macrophages was determined using flow cytometry. We demonstrate that RAW264.7 macrophages exhibit TRAIL-R1 surface expression and that the tested compounds: chrysin, apigenin and acacetin can inhibit TRAIL-R1 death receptor expression level on macrophages.

## 1. Introduction

Tumor Necrosis Factor-related apoptosis-inducing ligand (TRAIL) is a type II transmembrane protein (*N*-terminal located in the cell interior and *C*-terminal on the exterior) and is one of several members of the Tumor Necrosis Factor superfamily [1,2]. TRAIL mRNA can be detected in most human tissues, with significantly higher expression in the prostate and spleen. Additionally, TRAIL is expressed on the cell membrane of natural killer (NK) and T cells, monocytes/macrophages, and dendritic cells and can be cleaved into a soluble, secreted form [1,3,4,5,6,7]. The main function of TRAIL is induction of apoptosis in transformed, but not in normal cells. TRAIL induces apoptosis through engagement of death receptors. The TRAIL receptor family consists of four distinct membrane-bound receptors. Two of these—TRAIL-R1 (DR4, Apo2A, TNFRS10A) and TRAIL-R2 (DR5, KILLER, TRICK2, TNFRSF10B) are death receptors. Like all death receptors TRAIL-R1 and TRAIL-R2 contain a death domain, which is critically required for induction of programmed cell death. In contrast, TRAIL-R3 (DcR1, TRIDD, LIT), which lacks an intracellular death domain and TRAIL-R4 (DcR2, TRUNDD), which contains a truncated non-functional death domain, are unable to initiate apoptotic signaling and act as decoy receptors. The fifth TRAIL-binding receptor is osteoprotegerin (OPG), which is a soluble protein. OPG also binds another member of the tumor necrosis factor (TNF) family, TRANCE/RANKL. TRAIL-R1, -R2, -R3 and -R4 all map to a tight cluster on chromosome 8p21, suggesting that these genes evolved via duplication events from a common precursor [8,9,10,11,12,13,14,15,16,17,18].

**Figure 1:**
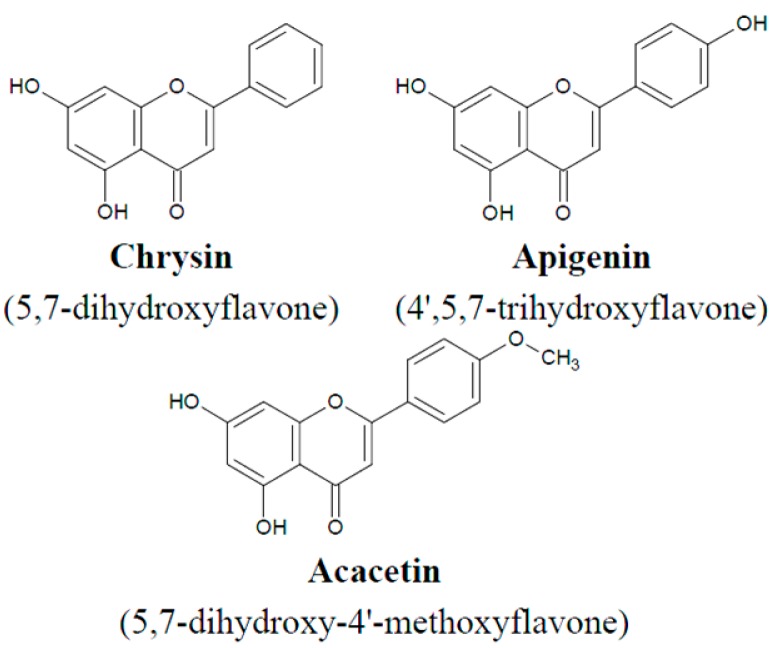
Chemical structure of tested flavones.

Macrophages are involved in both innate and adaptative immune responses, as phagocytes, antigen presenting cells and as effector cells of delayed-type hypersensitivity [19]. Activated macrophages undergo many changes that allow them to kill invading bacteria or infected cells. They release toxic chemicals and proteins (e.g., reactive oxygen species (ROS), reactive nitrogen species (RNS), and cytokines), which have toxic effects on other cells. There are two pathways of macrophage activation. Macrophages became classically activated by exposure to two signals: lipopolysaccharide (LPS) and interferon-gamma (IFN-γ). Classically activated macrophages exhibit a Th1-like phenotype, promoting inflammation, extracellular matrix (ECM) destruction, and apoptosis, while alternatively activated macrophages display a Th2-like phenotype, promoting ECM construction, cell proliferation, and angiogenesis [20,21,22]. Macrophage cells have surface signaling molecules and receptors, which determine their role in immunological reactions. There are no data about TRAIL receptor expression on macrophages however, and the aim of this study was to investigate the expression levels of TRAIL-R1 and TRAIL-R2 receptors on non-stimulated and LPS-stimulated macrophages form the cell line RAW264.7. Three substances belonging to flavonoids: chrysin, apigenin and acacetin, which differ in their substituents at the 4' position in phenyl ring were used in the assays due to the variety of biological activities of polyphenol compounds. Chrysin, apigenin and acacetin belong to flavones, a subclass of flavonoids naturally occurring in plants that have important biological activities. Some flavones have been found to posses various clinically relevant properties such as anti-tumor, anti-platelet, anti-inflammatory, antimicrobial, and antioxidant activity [23,24,25,26]. This study was designed to investigate the *in vitro* effect of chrysin, apigenin and acacetin (Figure 1) on TRAIL-R expression on the macrophage cell line RAW264.7.

## 2. Results and Discussion

Macrophages are immunocompetent cells, connecting an innate and an adaptive immune response [19]. Viability of RAW264.7 macrophages in the presence of LPS (200 ng/mL) and/or flavones (20 μM) was measured by MTT (3-(4,5-dimethyl-2-thiazyl)-2,5-diphenyl-2*H-*tetrazolium bromide )assay and LDH (lactate dehydrogenase) test. The results showed that LPS and tested flavones at tested concentration had no significantly effect on the viability and was not toxic to macrophages. There were not differences between control values and tested samples examined by LDH assay (data not shown).

**Figure 2:**
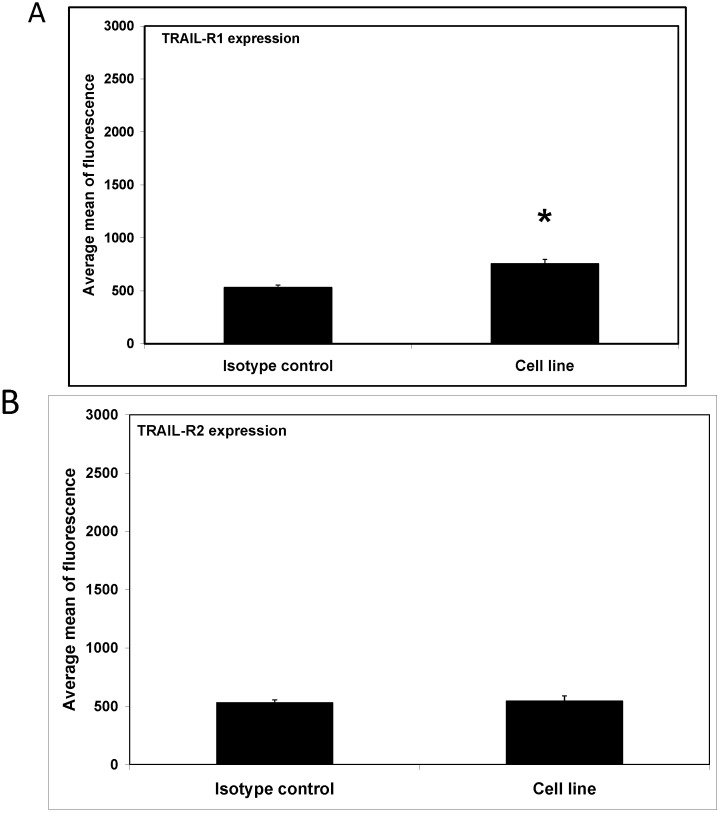
The surface expression of tumor necrosis factor-related apoptosis-inducing ligand (TRAIL) receptors TRAIL-R1 (**A**) and TRAIL-R2 (**B**) on macrophages RAW264.7, analyzed using flow cytometry. The values represent mean ± SD of three independent experiments. * *p* < 0.05 compared to isotype control.

One of the hallmarks of macrophages is their ability to respond to environmental stimuli and change their form and physiology. Macrophage cells have surface signaling molecules and receptors, which determine their role in immunological reactions, e.g., toll-like receptor (TLR), NOD-like receptor (NLR). Now we have tested for the first time the expression of TRAIL-R1 and TRAIL-R2 on RAW264.7 macrophages. The expression level of the TRAIL-R1 and TRAIL-R2 was examined by flow cytometry. Our results demonstrate that the macrophage cell line RAW264.7 has only the TRAIL-R1 receptor on its surface (Figure 2).

**Figure 3:**
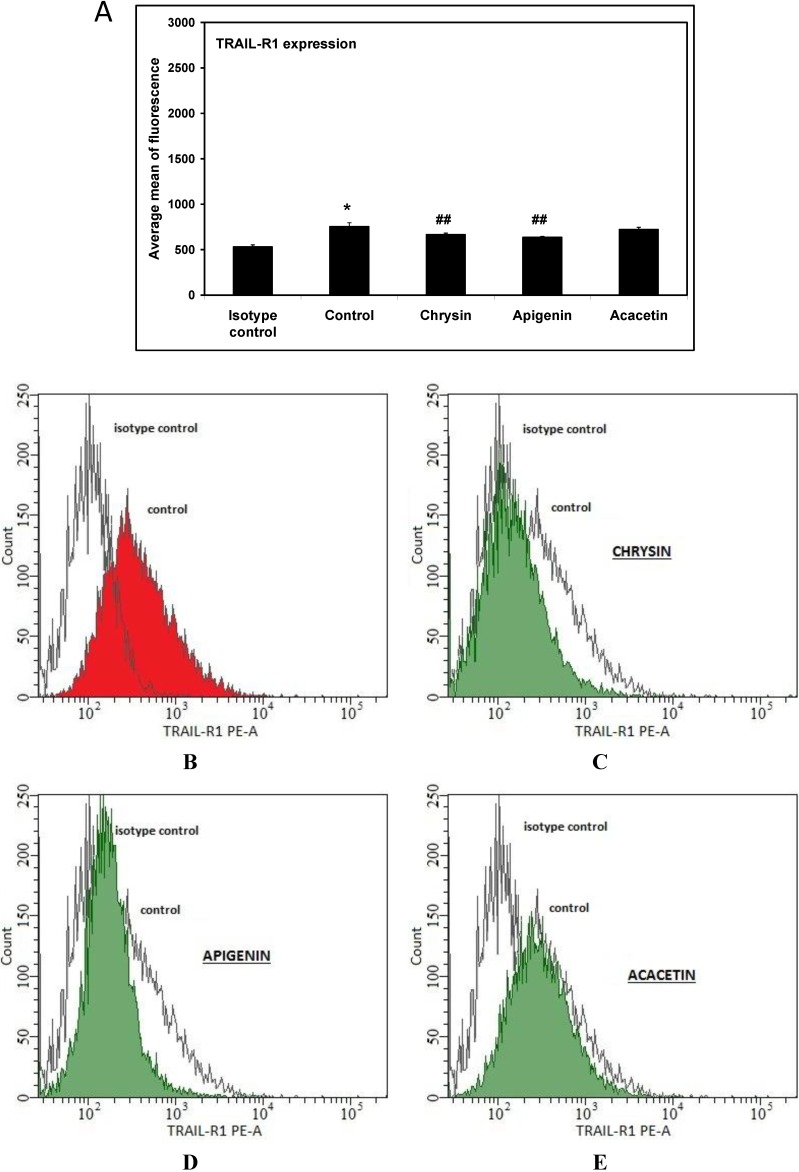
(**A**) Effects of flavones on TRAIL-R1 expression in RAW264.7 cells. Cells were incubated for 24 h alone (**B**) with chrysin (**C**), apigenin (**D**) and acacetin (**E**) at a concentration of 20 μM. The surface expression of TRAIL-R1 death receptors was measured by flow cytometric analysis. Representative histograms depict the average mean of fluorescence from three independent experiments performed in duplicate *n* = 6. * *p* < 0.05 compared to isotype control. **^##^** *p* < 0.01 compared to control.

After estimation of expression level of the TRAIL-R1 and TRAIL-R2 on the surface of RAW264.7 cells, we determined the expression of TRAIL-R1 and TRAIL-R2 after a 24-h incubation of macrophage cells with flavones at the concentration of 20 μM by flow cytometry. The previously obtained results showed that flavones at the tested concentration had no effect on viability and was not toxic to macrophages. At concentrations of 50 μM chrysin had cytotoxic effects on RAW264.7 cells, that excluded the increase concentration of tested compounds, and we used one, lower concentration for all compounds (data not shown). After treatment with acacetin, the expression level of TRAIL-R1 death receptor in macrophage cells did not change compared to control. However chrysin and apigenin significantly decreased expression level of the TRAIL-R1 receptor, by a mean of 11% and 15.7% respectively (Figure 3).

**Figure 4:**
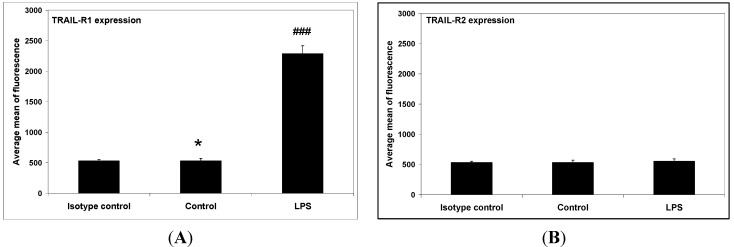
The surface expression of TRAIL-R1 (**A**) and TRAIL-R2 (**B**) on LPS (lipopolysaccharide)-activated macrophages RAW264.7, analyzed using flow cytometry. The values represent mean ± SD of three independent experiments. * *p* < 0.05 compared to isotype control. **^###^** *p* < 0.001 compared to control.

Next, the expression level TRAIL-R1 and TRAIL-R2 death receptors on LPS-activated macrophages (LPS; 200 ng/mL) was examined. LPS was found to significantly increase TRAIL-R1 death receptor expression, and additionally had no effect of TRAIL-R2 death receptor expression levels (Figure 4).

**Figure 5:**
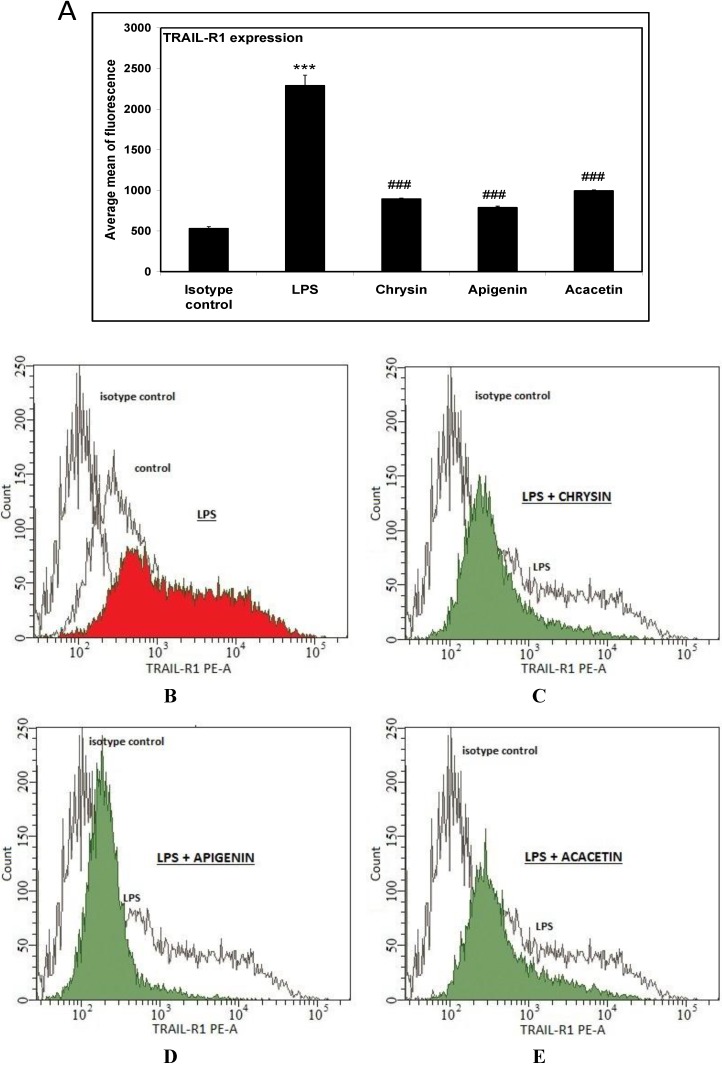
(**A**) Effects of flavones on TRAIL-R1 expression in RAW264.7 cells; Cells were incubated with LPS (200 ng/mL) (**B**); with LPS and chrysin (**C**); apigenin (**D**) and acacetin (**E**) at concentration of 20 μM. The surface expression of TRAIL-R1 death receptor was measured by flow cytometric analysis. Representative histograms depict the average mean of fluorescence from three independent experiments performed in duplicate *n* = 6. *** *p* < 0.001 compared to isotype control. **^###^** *p* < 0.001 compared to LPS-stimulated cells.

TRAIL-R1 and TRAIL-R2 expresson after treatment of macrophage cells with flavones at the concentration of 20 μM by flow cytometry was then determined. The results showed that all tested flavones significantly reduced levels of TRAIL-R1 expression on LPS-activated cells by a mean of 61% (for chrysin), 65.6% (for apigenin) and 56.6% (for acacetin) (Figure 5). Additionally none of the tested substances had an effect on TRAIL-R2 expression in LPS-activated cells (data not shown).

There are no data about TRAIL-R expression levels on murine macrophages RAW264.7. Wu *et al.*, (1999) [27] for the first time reported the isolation and characterization of a cDNA encoding the mouse receptor (MK) for TRAIL in mouse fibroblast NIH3T3 and the mouse embryonal carcinoma cell line F9. Like the human proapoptotic TRAIL receptors DR4 and KILLER/DR5, the mouse TRAIL receptor MK displays all the features characteristic of the tumor necrosis factor receptor (TNFR) family, and shows structural similarity to human TRAIL-R1 and TRAIL-R2 (79% and 76% respectively). Schneider *et al.*, (2003) [28] described two additional mouse receptors mDcR1 and DcR2, which are unable to initiate apoptotic signaling. However, most authors have demonstrated TRAIL-R expression in TRAIL-resistance human cancer cells. Horinaka *et al.*, (2005, 2006) [29,30] have demonstrated that apoptosis by luteolin and apigenin is mediated through death receptor 5 (DR5) upregulation and luteolin markedly induced the expression of TRAIL-R2 in human malignant tumor cells. Oishi *et al.*, (2013) [31] showed that the dietary flavonoid—apigenin binds and inhibits adenine nucleotide translocase-2 (ANT2), resulting in an enhancement of TRAIL-induced apoptosis by upregulation of TRAIL-R2 in the cancer cell line DU145 and LNCaP. Additionally the authors demonstrated that the isoflavone genistein did not upregulate TRAIL-R2 expression in prostate cancer cells by binding ANT2. Kim *et al.*, (2013) [32] have found that extracellular-signal regulated kinase (ERK) activation is involved in the induction of TRAIL-R2 expression in the cancer cell line HepG2. Inhibition of ERK1/2 significantly decreased the apigenin/TRAIL-induced TRAIL-R2 expression, what indicates that apigenin can enhance the apoptotic effect of TRAIL via ERK-induced upregulation of TRAIL-R2. Son *et al.*, (2007) [33] showed that silibinin sensitizes human glioma cells to TRAIL-mediated apoptosis via TRAIL-R2 upregulation. Taniquchi *et al.* (2008) [34] showed that the combination of baicalein and TRAIL effectively induced apoptosis in TRAIL-resistant colon cancer SW480 cells and the sensitivity was mediated through TRAIL-R2 upregulation. Here we demonstrate for the first time that RAW264.7 cells have only TRAIL-R1 surface expression and found that chrysin, apigenin and acacetin can inhibit TRAIL-R1 death receptor expression level on the macrophage surface; however, the mechanism of this inhibition is not yet clear.

**Figure 6:**
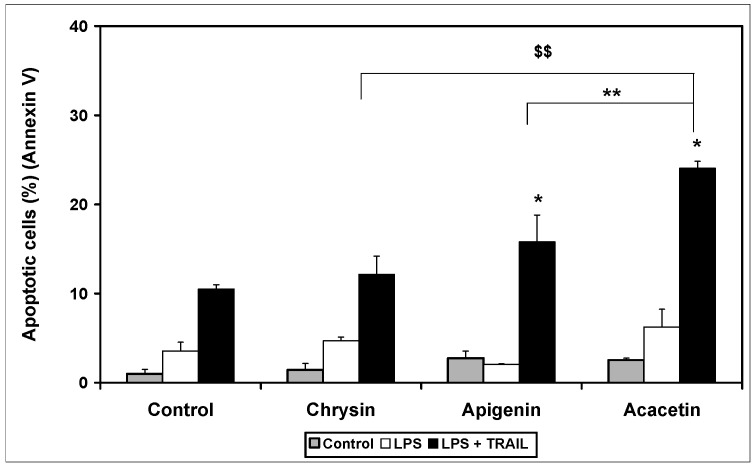
Apoptotic activity of TRAIL in combination with flavones in RAW264.7 cells. The macrophages were incubated for 24 h with TRAIL (100 ng/mL) and chrysin (20 μM), apigenin (20 μM) and acacetin (20 μM). Detection of apoptotic cell death by annexin V-FITC staining using flow cytometry.* *p* < 0.05; ** *p* < 0.01; ^$$^ *p* < 0.01 compared to chrysin.

The main role of the TRAIL cytokine is induction of apoptosis in cancer and transformed cells, not in normal cells. We examined the cytotoxic effect of TRAIL alone and in combination with tested flavones on RAW264.7 macrophages. Apoptotic cells were determined using flow cytometry. Tested flavones did not have cytotoxic effects on non-stimulated and LPS-stimulated macrophages compared to controls (Figure 6). Only TRAIL at a concentration of 100 ng/mL induced a minor apoptotic effect in RAW264.7 cells (10.5% ± 0.5%). Moreover, apigenin and acacetin in combination with TRAIL increased the percentage of apoptotic cells (15.8% ± 1.2% for apigenin and 27.1% ± 3.5% for acacetin). The apoptotic effect of TRAIL in combination with flavones is shown in Figure 6.

The expression level of TRAIL death and decoy receptors appears to be the most important factor of TRAIL-mediated apoptosis. Apoptotic assays using flow cytometry demonstrated that apigenin and acacetin augment TRAIL-mediated programmed cell death, in spite of inhibiting TRAIL-R1 expression on macrophages. Other authors have shown that polyphenols enhanced TRAIL-mediated apoptotic pathway signaling in cancer cells and sensitized resistant cancer cells to TRAIL. The mechanisms of the apoptotic and cytotoxic effects of flavones in combination with TRAIL on cancer cells have been described previously. Resistance to TRAIL in cells can be overcome by various molecular targets and was described previously [26,33,35,36]. 

## 3. Experimental Section

### 3.1. Cell Culture

Murine macrophage cell line RAW264.7 (established from a tumor induced by Abelson murine leukemia virus) was obtained from the ATCC (American Type Culture Collection, Manassas, VA, USA). The cells were cultured in Dulbecco’s modified Eagle’s medium (DMEM) supplemented with 10% heat inactivated fetal bovine serum (FBS, 10%), 4 mM l-glutamine, 100 U/mL penicillin, and 100 μg/mL streptomycin and maintained in monolayer cultures at the temperature 37 °C and atmosphere containing 5% CO_2_. Reagents for cell culture were purchased from ATCC. RAW264.7 were seeded at a density of 1 × 10^6^ cells/mL (2 × 10^5^ cells/well) in 96-well plates with or without LPS (200 ng/mL) and with or without flavones for 24 h [37,38].

### 3.2. Flavones

Chrysin, apigenin and acacetin were obtained from Sigma Chemical Company (Buchs, Switzerland). The compounds were dissolved in dimethyl sulphoxide (DMSO) to obtain the working concentration solutions. Lipopolysaccharide (LPS *E.* *coli* O:111:B4) was purchased from Sigma Chemical Company, (St. Louis, MO, USA).

### 3.3. Cell Viability

Cell viability was determined by 3-(4,5-dimethyl-2-thiazyl)-2,5-diphenyl-2H*-*tetrazolium bromide (MTT) reduction assay as previously described [37,39]. The RAW264.7 cells (1 × 10^6^ cells/mL) were seeded 3 h before the experiments in a 96-well plate. The macrophages RAW264.7 cells (1 × 10^6^ cells/mL) were treated with flavones (20 μM) with or without LPS (200 ng/mL) for 24 h. Final volume was 200 μL. Next the medium was removed, and MTT solutions (20 μL, 5 mg/mL; Sigma Chemical Company, St. Louis, MO, USA) were added to each well for 2 h. The resulting formazan crystals were dissolved in DMSO (100%). Controls included native cells and medium alone. The spectrophotometric absorbance at 550 nm was measured using an ELx 800 microplate reader (Bio-Tek Instruments Inc., Winooski, VT, USA). The viability was calculated by the formula: percent of viable cells = (absorbance of experimental wells/absorbance of control wells) × 100%.

### 3.4. Lactate Dehydrogenase Release Assay

Lactate dehydrogenase (LDH) is a stable cytosolic enzyme released upon membrane damage in necrotic cells. LDH activity was measured using a commercial cytotoxity assay kit (Roche Diagnostics GmbH, Mannheim, Germany). The RAW264.7 cells (1 × 10^6^ cells/mL) were treated with flavones (20 μM) with or without LPS for the indicated period of time. LDH released in culture supernatants is detected with coupled enzymatic assay, resulting in the conversion of a tetrazolium salt into a red formazan product. The maximal release was obtained after treating control cells with 1% Triton X-100 (Sigma Chemical Company) for 10 min at room temperature. The spectrophotometric absorbance at 490 nm was measured using the ELx 800 microplate reader. The percentage of cytotoxic cells was calculated using the formula: (sample value/maximal release) × 100% [37,39].

### 3.5. Flow Cytometric Analysis of Death Receptor Expression on the RAW264.7 Cells

The cell surface expression of TRAIL-R1 and TRAIL-R2 death receptors was detected by flow cytometry (LSR II Flow Cytometer; BD Biosciences, San Jose, CA, USA). RAW264.7 macrophages (2.5 × 10^5^ cells/mL) were seeded in 24-well plates for 24 h and exposed to flavones (20 μM) for 24 h. Cells were then harvested mechanically, washed twice with PBS (phosphate buffered saline) and resuspended in PBS containing 0.5% bovine serum albumin. Cells were incubated with 10 μL phycoerythrin-conjugated anti-TRAIL-R1 or anti-TRAIL-R2 monoclonal antibodies (R&D Systems, Minneapolis, MN, USA) at 4 °C for 60 min. After staining, the cells were washed with PBS and analysed using flow cytometry. The number of counted cells was 10^4^. The control sample (isotype control) consisted of cells in a separate tube treated with phycoerythrin-labelled mouse IgG1 or mouse IgG2B (R&D Systems, Minneapolis, MN, USA). Final volume of samples was 400 μL [35].

### 3.6. Detection of Apoptosis by Flow Cytometry

Apoptosis was measured using flow cytometry to quantify the levels of phosphatidylserine (PS) on the outer membrane of apoptotic cells. Externalized PS on the outer surface of the cytoplasmic membrane becomes labelled by Annexin V-FITC, which has a high affinity for PS-containing phospholipid bilayers. The Annexin V assay was performed using the Apoptotest™-FITC Kit (Dako, Glostrup, Denmark). RAW264.7 macrophages (2 × 10^5^ cells/mL) were seeded in 24-well plates for 24 h and then exposed to flavones (20 μM) and/or TRAIL (20–100 ng/mL) for 24 h. After this time RAW264.7 cells were washed twice with PBS and resuspended in binding buffer (0.5 mL). The cell suspension was then incubated with Annexin V-FITC (5 μL) and propidium iodide (PI, 5 μL) for 10 min at room temperature in the dark. The population of Annexin V-positive cells was evaluated by flow cytometry (LSR II Flow Cytometer; BD Biosciences, San Jose, CA, USA). The number of counted cells was 10^4^ [35,36].

## 4. Conclusions

Our study indicated that RAW264.7 macrophages express TRAIL-R1 but not TRAIL-R2. Moreover, tested flavones (chrysin, apigenin and acacetin) can inhibit TRAIL-R1 expression on non-stimulated and LPS-stimulated macrophages. In spite of inhibiting TRAIL-R1 expression level on macrophages, some tested flavones such as apigenin and acacetin enhanced TRAIL-mediated apoptosis.
